# Taxonogenomics reveal multiple novel genomospecies associated with clinical isolates of *Stenotrophomonas maltophilia*

**DOI:** 10.1099/mgen.0.000207

**Published:** 2018-08-07

**Authors:** Prashant P. Patil, Sanjeet Kumar, Samriti Midha, Vikas Gautam, Prabhu B. Patil

**Affiliations:** ^1^​ Bacterial Genomics and Evolution Laboratory, CSIR–Institute of Microbial Technology (CSIR-IMTECH), Chandigarh, India; ^2^​ Department of Medical Microbiology, Post Graduate Institute of Medical Education and Research (PGIMER), Chandigarh, India; ^†^​ Present address: Institute of Infection and Global Health, University of Liverpool, Liverpool, UK.

**Keywords:** *Stenotrophomonas maltophilia*, genomospecies, taxonogenomics, resistome, phylogenomics

## Abstract

*Stenotrophomonas maltophilia* has evolved as one of the leading multidrug-resistant pathogens responsible for a variety of nosocomial infections especially in highly debilitated patients. As information on the genomic and intraspecies diversity of this clinically important pathogen is limited, we sequenced the whole genome of 27 clinical isolates from hospitalized patients. Phylogenomic analysis along with the genomes of type strains suggested that the clinical isolates are distributed over the *Stenotrophomonas maltophilia* complex (Smc) within the genus *Stenotrophomonas*. Further genome-based taxonomy coupled with the genomes of type strains of the genus *Stenotrophomonas* allowed us to identify five cryptic genomospecies, which are associated with the clinical isolates of *S. maltophilia* and are potentially novel species. These isolates share a very small core genome that implies a high level of genetic diversity within the isolates. Recombination analysis of core genomes revealed that the impact of recombination is more than mutation in the diversification of clinical *S. maltophilia* isolates. Distribution analysis of well-characterized antibiotic-resistance and efflux pump genes of *S. maltophilia* across multiple novel genomospecies provided insights into its antibiotic-resistant ability. This study supports the existence of multiple cryptic species within the Smc besides *S. maltophilia*, which are associated with human infections, and highlights the importance of genome-based approaches to delineate bacterial species. This data will aid in improving clinical diagnosis and for understanding species-specific clinical manifestations of infection due to *Stenotrophomonas* species.

## Data Summary

1. The draft genome assembly of 27 clinical isolates of *S. maltophilia* under this study have been deposited in GenBank and individual accession numbers are provided in Table 1.

2. Phylogenetic tree file, i.e. Newick file (.nwk), generated from maximum-likelihood reconstruction based on concatenation of protein sequence from 23 phylogenomic reference genes of 27 *S. maltophilia* clinical isolates and type strains of the genus *Stenotrophomonas* are deposited in Figshare; DOI:10.6084/m9.figshare.5356132 (https://figshare.com/s/2efe1ba9e515343e5017).

3. Phylogenetic tree file, i.e. Newick file (.nwk), for a robust phylogenetic tree based on the alignment of protein sequences from 400 core genes of 27 *S. maltophilia* clinical isolates under study along with the type strains of members of the *Stenotrophomonas maltophilia* complex are deposited in Figshare; DOI:10.6084/m9.figshare.5356156 (https://figshare.com/s/2db426f19b14a6706e43).

4. Data file (.xlsx) used to generate the heat map of average nucleotide identity (ANI) values of *S. maltophilia* clinical isolates with the type strains of species belonging to the genus *Stenotrophomonas* is deposited in Figshare; https://figshare.com/s/dc32d3b7be5f18012fbb.

5. Data file (.xlsx) used to generate the heat map of digital DNA–DNA hybridization (dDDH) values of *S. maltophilia* clinical isolates with the type strains of species belonging to the genus *Stenotrophomonas* is deposited in Figshare; https://figshare.com/s/8c9dc8b9e76661984d92.

6. Data file (.csv) used to generate the heatmap of presence and absence of antimicrobial resistance genes is deposited in Figshare; DOI:10.6084/m9.figshare.5353696 (https://figshare.com/s/af35f8952e15bd07dce1).

**Impact Statement**

*Stenotrophomonas maltophilia* is a rapidly emerging multi-drug-resistant opportunistic pathogen responsible for nosocomial infections and a serious threat to healthcare settings worldwide. The genus *Stenotrophomonas* is taxonomically challenging due to several reclassifications and misclassifications associated with it. Genotypic methods suggest a high level of genetic diversity among *S. maltophilia* isolates. Thus, there is a need to assess the intra-species diversity of *S. maltophilia* among clinical isolates and to delineate them to the correct species. Type strains of the genus *Stenotrophomonas* are now available in public databases. Thus, we assessed intra-species diversity among clinical isolates of *S. maltophilia* by genome sequencing and integrated them with the genomes of type strains of the genus *Stenotrophomonas* using modern taxonomic methods. This allowed us to delineate clinical isolates within the genus and discover potential novel species of the genus responsible for clinical infections. We also studied the contribution of point mutations, homologous recombination and horizontal gene transfer in the diversification of clinical *S. maltophilia* isolates. Our finding of potential novel species of *Stenotrophomonas* associated with human infections may open up a new path for further studies on the epidemiology, disease spectrum, virulence and resistance traits of infections.

## Introduction

The genus *Stenotrophomonas* currently comprises 13 validated species according to the List of Prokaryotic Names with Standing in Nomenclature (LPSN; http://www.bacterio.net), which are versatile and have the ability to adapt to diverse environmental niches [1, 2]. *Stenotrophomonas maltophilia* is an important and predominant species of the genus *Stenotrophomonas* with a wide range of activities, including plant growth promotion, breakdown of man-made pollutants and production of secondary metabolites, and it has an improtant role in multi-drug-resistant infections to humans and animals [2–4]. *S. maltophilia* is a multi-drug-resistant opportunistic pathogen responsible for causing infections in hospitalized patients as well as cystic fibrosis and cancer patients [5–8]. According to a recent World Health Organization report, *S. maltophilia* is one of the leading multi-drug-resistant bacteria in healthcare settings worldwide [9].

The taxonomic status of *S. maltophilia* within the genus is complicated because several previously proposed species, namely *S. africana*, *Pseudomonas genicualata*, *Pseudomonas hibiscicola* and *Pseudomonas beteli*, are considered as synonyms of *S. maltophilia* [10]. *S. maltophilia* and its synonym species along with the validly described *Stenotrophomonas pavanii* belong to the *Stenotrophomonas maltophilia* complex (Smc) [11, 12]. Whole-genome sequencing of the type strains of validly described and misclassified species belonging to the genus *Stenotrophomonas* revealed that synonyms of *S. maltophilia*, i.e. *S. africana*, *P. genicualata*, *P. hibiscicola* and *P. beteli*, represent distinct species as per modern genome-based taxonomic criteria [13]. In addition to this taxonomic complication, clinical and environmental isolates of *S. maltophilia* exhibit high levels of phenotypic and genotypic diversity [14]. Various molecular typing methods such as amplified fragment length polymorphism (AFLP) [1], rep-PCR [15], *gyrB* [10] and multi-locus sequence typing and analysis [16–18] have shown that there is a high level of genetic diversity amongst *S. maltophilia* isolates. Although these approaches have provided insights into the phylogeny and genetic diversity among *S. maltophilia* isolates, their limited resolution at the strain level means they are not useful for studies of intraspecies diversity. Genomic studies of clinical and environmental *S. maltophilia* isolates also suggested a high level of genomic diversity among them [19–23], but systematic studies focusing on phylogenomics and taxogenomics are lacking. Thus, there is a need to understand the intraspecies diversity of *S. maltophilia* clinical isolates by using genome-based approaches, which is important to identify novel species associated with human infections.

Sequencing of a clinical strain, K279a, of *S. maltophilia* revealed that the presence of numerous drug resistance determinants and efflux pumps into its genome [24]. *S. maltophilia* is resistant to a broad array of antibiotics due to intrinsic resistance mechanisms, which are common to all *S. maltophilia* isolates. Such resistance mechanisms include low membrane permeability, the presence of efflux pumps and antibiotic-modifying enzymes [7, 25]. The intrinsic resistome includes chromosomal but not horizontally acquired genes, which are present in all strains of bacterial species prior to antibiotic exposure. Moreover, apart from the intrinsic resistance mechanisms, acquired mechanisms have also been reported in *S. maltophilia*, involving acquisition of resistance genes through horizontal gene transfer and mutations [26, 27].

In the present study, whole genome sequencing of 27 clinical isolates identified as *S. maltophilia* isolated from hospitalized patients at the Postgraduate Institute of Medical Education and Research (PGIMER), Chandigarh, India, was carried out. To study phylogenetic placements of sequenced clinical isolates within the genus *Stenotrophomonas* and to discover novel genomospecies, we used type strain-based phylogenomics and modern taxonomic criteria. Based on this, we concluded that multiple novel genomospecies are present amongst these clinical isolates of *S. maltophilia.* We also studied the gene content of Smc members along with novel genomospecies and found a small core genome size, which again supported the diverse nature of these clinical isolates. To elucidate the role of homologous recombination and mutations in the diversification of the Smc, we performed recombination analysis, which suggested that the impact of homologous recombination includes more than mutations in diversification. We also assessed the distributions of drug resistance and efflux pump genes across novel genomospecies. Our finding of potential novel species associated with the clinical isolates of *S. maltophilia* may be important for clinicians in understanding the epidemiology and management of the disease caused by this multi-drug-resistant pathogen.

## Methods

### Bacterial isolates and culture conditions

Twenty-seven isolates identified as *S. maltophilia* from hospitalized patients at a tertiary care hospital, PGIMER, were included in this study (Table 1). They were isolated from different clinical specimens, i.e. blood (*n*=18), respiratory (*n*=7), pus (*n*=1) and cerebrospinal fluid (*n*=1). The isolates were grown either on nutrient agar or in nutrient broth at 37 °C from frozen stocks. Ethics approval and each patient’s written consent was not required as it was a part of routine clinical testing.

**Table 1.** List of whole genome sequenced clinical isolates of *S. maltophilia*, their isolation source, genome features, contamination and completeness estimates along with NCBI accession

	Isolate ID	Source	Year	Genome size (bp)	No. of contigs	Fold coverage	N50 (bp)	% GC	No. of CDS*	Total bp in reads	Completeness (CheckM)	Contamination (CheckM)	NCBI accession
1	SM20065	Blood	2012	4 503 178	137	266	57 893	66.5	3965	1200729721	99.01	0.00	LXXA00000000
2	SM3226	Blood	2012	4 485 531	181	157	44 537	66.6	3910	706934712	99.89	0.18	LXXB00000000
3	SM325416	Blood	2013	4 499 756	166	234	67 358	66.6	3967	1054601188	99.15	0.03	LXXC00000000
4	SM7180	Respiratory	2012	4 559 054	164	239	62 326	66.5	3980	1092251589	99.66	0.00	LXXD00000000
5	SM7882	Respiratory	2012	4 404 542	143	407	62 986	66.5	3897	1793580861	97.93	0.69	LXXE00000000
6	SM480	Respiratory	2013	4 294 147	143	210	81 284	66.6	3741	905057243	95.63	0.34	LXXF00000000
7	SM11522	Blood	2012	4 772 386	163	238	66 742	66.2	4227	1138488981	95.62	0.69	LXXG00000000
8	SM2546	Respiratory	2013	4 622 345	145	267	64 336	64.5	4099	1238064180	98.62	0.39	LXXH00000000
9	SM4416	Blood	2012	4 348 678	254	133	42 949	66.8	3806	582189524	99.26	0.05	LXXI00000000
10	SM100	Blood	2010	4 670 638	224	65	38 440	66.4	4185	307302019	98.74	0.43	LXXJ00000000
11	SM19467	Blood	2012	4 590 360	162	201	54 275	66.5	4084	926278460	99.10	0.34	LXXK00000000
12	SM30540	Blood	2013	4 544 171	147	197	54 368	66.6	4042	898987912	99.31	0.00	LXXL00000000
13	SM5815	Blood	2010	4 927 374	247	125	48 474	66.4	4432	618830138	99.74	2.12	LXXM00000000
14	SM17711	Blood	2012	4 334 100	143	195	69 796	66.9	3803	846083847	98.62	0.00	LXXN00000000
15	SM24179	Blood	2012	4 281 782	140	252	76 600	66.9	3754	1082737078	95.66	0.00	LXXO00000000
16	SM6957	Blood	2013	4 300 278	128	245	74 488	66.7	3814	1054847302	98.26	0.00	LXXP00000000
17	SM1911	Pus	2010	4 279 279	230	67	47 434	66.7	3758	289442792	98.03	0.11	LXXQ00000000
18	SM13670	Blood	2012	4 316 491	136	240	62 147	66.6	3814	1038281752	98.14	0.17	LXXR00000000
19	SM760	Respiratory	2010	4 227 019	253	109	38 897	66.7	3681	461064721	97.75	0.11	LXYA00000000
20	SM1006	Blood	2013	4 308 146	119	339	76 996	66.6	3799	1464096438	98.83	0.00	LXXS00000000
21	SM3112	Respiratory	2012	4 272 442	150	207	69 629	66.6	3771	887873333	99.27	0.39	LXXT00000000
22	SM16975	Blood	2012	4 582 512	119	256	83 180	66.4	4093	1173248392	97.76	0.39	LXXZ00000000
23	SM10507	CSF†	2012	4 783 681	167	140	71 880	66.4	4296	671034607	99.80	1.03	LXXU00000000
24	SM16360	Blood	2012	4 712 691	116	258	96 011	66.6	4163	1219487828	98.62	1.49	LXXV00000000
25	SM1389	Blood	2010	4 350 701	122	314	64 824	66.5	3862	1369078280	98.03	0.00	LXXW00000000
26	SM38795	Blood	2013	4 227 221	136	245	79 994	66.6	3726	1039225345	96.16	0.34	LXXX00000000
27	SM3123	Respiratory	2010	4 018 348	114	215	138 936	66.9	3491	865543650	95.55	0.33	LXXY00000000

*CDS, coding DNA sequences.

†CSF, cerebrospinal fluid.

### DNA isolation, Illumina library construction and sequencing

Approximately 15 ml of culture was grown in nutrient broth at 37 °C with constant shaking at 200 r.p.m. DNA isolation was carried out by using a ZR Fungal*/*Bacterial DNA MiniPrep Kit (Zymo Research) as per the manufacturer’s instructions. DNA was quantified by using a Qubit 2.0 Fluorometer (Invitrogen; Thermo Fisher Scientific). Illumina sequencing libraries were prepared by using an Illumina Nextera XT sample preparation kit (Illumina) with dual indexing adapters from Illumina by strictly following the manufacturer's guidelines. Illumina libraries were quantified by using a KAPA Library Quantification kit for Illumina (KAPA Biosystems). Sequencing libraries were pooled and sequenced using an in-house Illumina Miseq (Illumina) platform with 2×250 bp paired-end runs.

### Genome assembly and annotation

The Illumina reads were *de novo* assembled into the high-quality draft genome by using CLC Genomics Workbench 6.5.1 (CLC Bio-Qiagen) with default parameters except a minimum contig length set to 500 bp. The quality of the assembled genome in terms of completeness and contamination was accessed using CheckM v1.0.7 with default settings [28]. The assembled genomes were submitted to the NCBI GenBank database and accession numbers are given in Table 1. The genomes were annotated using the NCBI-Prokaryotic genome annotation pipeline [29].

### Phylogenetic analysis

The 16S rRNA gene was extracted from the sequenced genome by using the RNAmmer 1.2 server [30] available at http://www.cbs.dtu.dk/services/RNAmmer/. Protein sequences for 23 essential bacterial phylogenetic reference genes (*dnaG*, *rplA*, *rplB*, *rplC*, *rplD*, *rplE*, *rplF*, *rplK*, *rplL*, *rplM*, *rplN*, *rplP*, *rplS*, *rpmA*, *rpoB*, *rpsB*, *rpsC*, *rpsE*, *rpsJ*, *rpsK*, *rpsM*, *rpsS*, *tsf*) were extracted from the genome by using the AmphoraNet pipeline [31] available at http://pitgroup.org/amphoranet/. The extracted sequences were aligned by using clustalW and a maximum-likelihood (ML) phylogenetic tree was reconstructed by using the General Time Reversible model, and Gamma distributed and Invariant sites (G+I) with 1000 bootstrap replications using mega version 6.06 [32]. The phylogenetic tree based on the whole genome was reconstructed by using PhyloPhlAn [33], which uses 400 ubiquitous and phylogenetically informative proteins conserved among the bacteria. Orthologues of these proteins in the genome were detected using usearch v5.2.32 [34] followed by the generation of multiple sequence alignments of these proteins using muscle v3.8.31. A final concatenated dataset containing 4231 aligned amino acid positions was generated, and phylogenetic tree reconstruction was performed using FastTree version 2.1. [35]. The resulting phylogenetic tree was visualized by using iTOL v4 (https://itol.embl.de/) [36].

### Genome similarity assessment

For genome similarity assessment we used average nucleotide identity (ANI) and digital DNA–DNA hybridization (dDDH), which have emerged as modern genome-based taxonomic methods [37]. ANI was calculated by using JSpecies 1.2.1 [38] and dDDH was calculated by using the web tool Genome to Genome Distance Calculator, GGDC 2.1 (http://ggdc.dsmz.de/distcalc2.php). We used Formula 2 alone for calculation of dDDH as it determines dDDH independent of the genome length and is recommended for use with draft genomes [39]. Heat maps of ANI and dDDH values were constructed using gene-e software (https://software.broadinstitute.org/GENE-E/).

### Pan-genome analysis

Pan and core genome analysis were performed using the pan-genome analysis pipeline (PGAP pipeline version 1.2.1) with the MultiParanoid (MP) method [40]. A minimum score value of 40 and e-value of 1e-10 were used as a cut-off for blast. PanGP version 1.0.1 [41] was used to analyse the pan-genome profile of clinical isolates of *S. maltophilia* and six reference genomes of members of the Smc. The power-law regression (*y*_pan_=*A*_pan_* x^B^*^pan^+*C*_pan_) was used to model the pan-genomes generated from all permutations, where *y*_pan_ is the total number of gene families in the pan-genome, *x* is the number of genomes considered, and *A*_pan_, *B*_pan_ and *C*_pan_ are fitting parameters. When 0<*B*_pan_<1, the pan-genome should be considered open because it is an unrestrained function over the number of genomes. When *B*_pan_<0, the pan-genome is considered closed because it approaches a constant as more genomes are considered. The number of core genes after addition of each new genome was plotted as a function of the number of genomes added sequentially, in a similar manner to the pan-genome plot. The exponential curve fit model, *y*_core_*=A*_core_* e^B^*^core^*^.x^+C*_core_, was used to fit the core genome, where *y*_core_ denotes the core genome size, *x* denotes the number of genomes, and *A*_core_, *B*_core_ and *C*_core_ are fitting parameters.

### Homologous recombination analysis

The genomes of clinical isolates of *S. maltophilia* along with the five type strains belonging to the Smc were aligned using Mauve version 20150226 build 10 (c) [42]. Core genome alignment generated from Mauve was further used to reconstruct the phylogenetic tree using PhyML 3.1 [43]. The core genome alignment and PhyML tree were further used to calculate the relative rate of recombination to mutation events using ClonalFrameML [44] with 100 bootstrap replications. The PhyML and ClonalFrameML phylogenetic tree was visualized by using iTOL v4 (https://itol.embl.de/) [36].

### Resistome analysis

The nucleotide sequences of well-characterized antibiotic resistance and efflux pump genes were retrieved from the complete genome of *S. maltophilia* K279a. The nucleotide sequences of resistant genes were used as query in blast v2.2.28+ [45] searches with sequenced genomes in order to check the distribution of drug resistance genes amongst diverse genomospecies of *S. maltophilia*. The heat map of presence and absence of resistance-associated genes was generated using gene-e software (https://software.broadinstitute.org/GENE-E/).

## Results

### Whole genome sequencing of *S. maltophilia* clinical isolates

Whole genome sequencing was carried out for *S. maltophilia* isolated during 2010–2013 from clinical specimens of different patients (Table 1). The genome features and assembly statistics are detailed in Table 1. High-quality draft genomes were obtained with coverage ranging from 65× to 407× fold. There is no direct significant correlation found between assembly quality and coverage, suggesting that other factors, such as library quality or percentage of repetitive DNA in each genome, may influence the assembly quality. The estimated genome completeness for this genome dataset ranges from 95.55 to 99.89 % and estimated contamination ranges from 0 to 2.12 % (Table 1). The number of predicted coding DNA sequences (CDSs) ranged from 3491 to 4432 and GC content of the assembled genomes is around 66  mol% (Table 1).

### Phylogenetic placement of sequenced clinical *S. maltophilia* isolates within the genus *Stenotrophomonas*

**Fig. 1.**
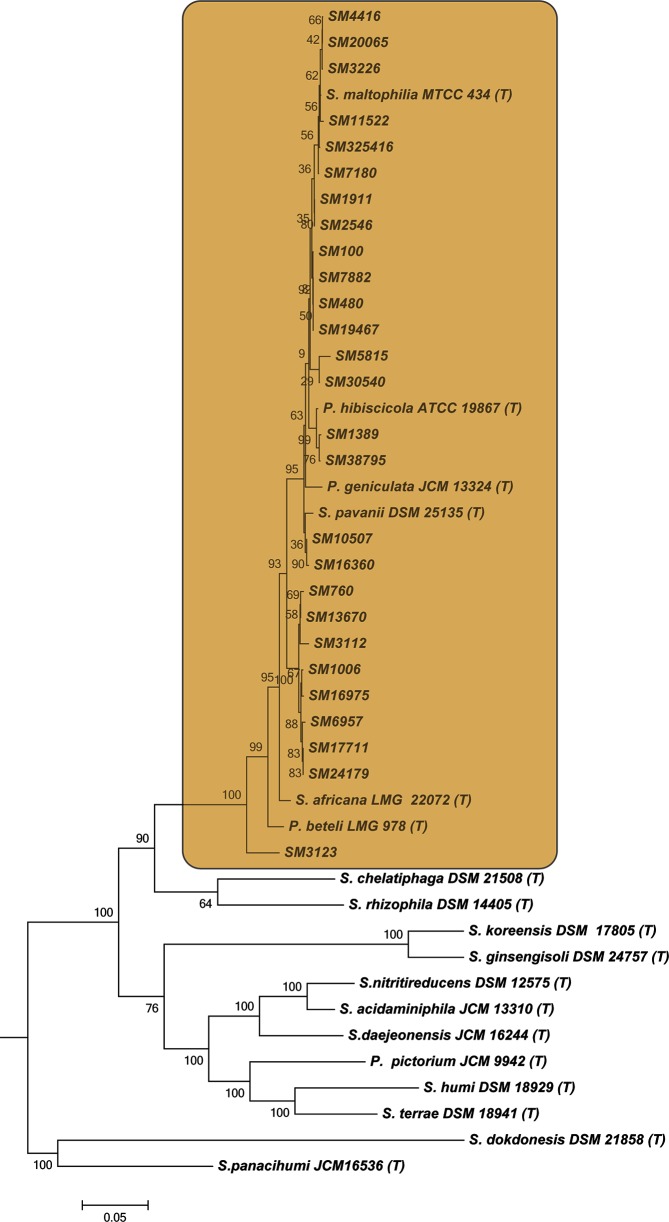
Phylogenetic placement of *S. maltophilia* clinical isolates within the genus *Stenotrophomonas*. ML reconstruction based on concatenation of translated protein sequences of 23 phylogenomic reference genes of clinical isolates and the type strains within the genus *Stenotrophomonas.* Bar (0.05), the number of amino acid substitutions per site. The phylogenetic clade representing the *Stenotrophomonas maltophilia* complex is highlighted. Bootstrap values shown at nodes are the percentage of 1000 replicates.

The phylogenetic placement of sequenced clinical *S. maltophilia* isolates within the genus *Stenotrophomonas* was assessed by reconstructing a phylogenetic tree along with the type strains of species belonging to the genus (Table S1, available in the online version of this article). A phylogenetic tree was reconstructed based on 16S rRNA gene sequences, which plays an important role in microbial identification and taxonomy with 97 % cut-off for distinct species. 16S rRNA gene sequences of the clinical isolates from this study showed >97 % similarity with the type strains of all species of the Smc (Fig. S1). Due to the limited resolution provided by 16S rRNA-based phylogeny, a phylogenomic tree was obtained further using translated protein sequences of 23 conserved housekeeping genes. The analysis showed the placement of *S. maltophilia* clinical isolates in the Smc with high bootstrap values (Fig. 1). While both analyses suggested the distribution of clinical isolates over the Smc lineage, certain discrepancies in branching among the phylogenetic tree based on 16S rRNA and 23 phylogenomic marker genes were observed, indicating the need for a highly robust tree for taxonomic classification.

**Fig. 2.**
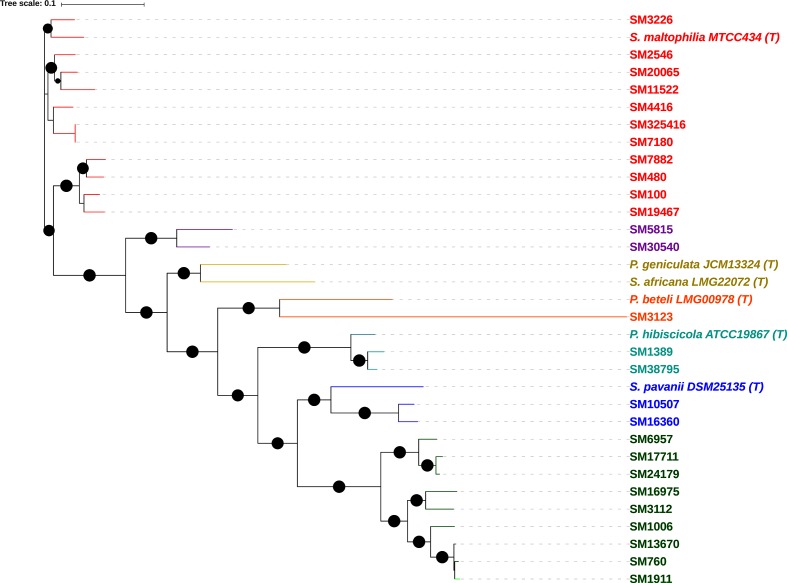
Phylogenetic tree based on protein sequences of 400 core genes of 27 *S. maltophilia* clinical isolates under study and the type strains of members of the Smc. Nodes overlaid with a black dot represent a bootstrap value of >95 %. Different highlighted colours represent different clades. Species type strains are marked (T). Bar (0.1), the number of amino acid substitutions per site.

To address these discrepancies, we reconstructed a phylogenetic tree based on protein sequences of 400 core genes of the Smc including type strains of members of the Smc along with *S. maltophilia* clinical isolates under study (Fig. 2). The phylogenetic tree showed that the 27 clinical isolates of *S. maltophilia* were distributed over five major monophyletic groups. Eleven isolates grouped together with *S. maltophilia* MTCC 434^T^ while both *P. hibisicola* ATCC 19867^T^ and *S. pavanii* DSM 25135^T^ were grouped with two isolates under study. Isolate SM3123 formed a monophyletic clade with *P. beteli* LMG 978^T^. The type strains of *P. geniculata* and *S. africana* did not group with any of the clinical isolates under study.

### Genome similarity assessment and discovery of novel genomospecies

**Fig. 3.**
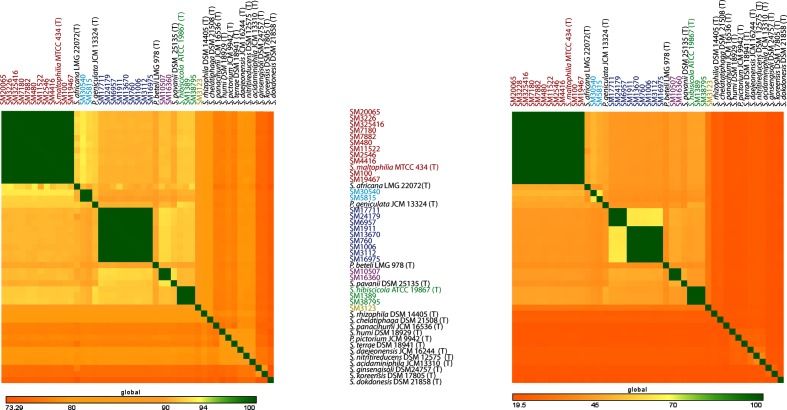
Heatmap of ANI and dDDH values among 27 clinical isolates with the type strains of members of the genus *Stenotrophomonas* under species delineation thresholds. The left side heat map represents ANI and the right side dDDH values. Colour variation in heat maps shows the variation in identity values as shown by the scale on the bottom. Isolate names highlighted with the same colour belong to the same genomospecies, and those that do not group with any isolate under study are highlighted as black.

A robust phylogenetic tree of members of the Smc clearly revealed the existence of multiple distinct lineages within the Smc. We calculated ANI and dDDH values with the type strains of valid and misclassified species of the genus *Stenotrophomonas* for the assessment of overall genome similarity and to identify potential novel species. The heat map of ANI and dDDH values of clinical isolates of *S. maltophilia* with the type strains of the genus *Stenotrophomonas* is shown in Fig. 3. Based on the cut-off values for species delineation using ANI (96 %) and dDDH (70 %) [46], there are six distinct groups in *S. maltophilia* isolates that should be considered as separate bacterial species, and referred to below as genomospecies (Fig. 3). Genomospecies 1 (G1) consisting of 11 isolates that are grouped with reference strain *S. maltophilia* MTCC 434^T^ represents the core *S. maltophilia* group. Genomospecies 2 (G2), genomospecies 3 (G3) and genomospecies 4 (G4) comprised two, nine and two isolates, respectively, which did not group with any *Stenotrophomonas* species type strain (Fig. 3). Genomospecies 5 (G5) included two isolates that grouped with *P. hibiscicola* ATCC 19867^T^. Isolate SM3123 was a singlet as it did not group with any type strain within the genus *Stenotrophomonas* and is represented as genomospecies 6 (G6). The genome similarity results for the 27 Smc clinical isolates revealed their distribution over six genomospecies, among which G1 belongs to *S. maltophilia* and the remaining genomospecies (G2–G6) are potentially novel species (Table 2).

**Table 2.** List of genomospecies identified among 27 clinical isolates of *S. maltophilia* along with their species status

Genomospecies	Species	Isolates
Genomospecies 1 (G1)	*S. maltophilia*	SM20065, SM3226, SM325416, SM7180, SM7882, SM480, SM11522, SM2546, SM4416, SM100, SM19467
Genomospecies 2 (G2)	Novel	SM30540, SM5815
Genomospecies 3 (G3)	Novel	SM17711, SM24179, SM6957, SM1911, SM13670, SM760, SM1006, SM3112, SM16975
Genomospecies 4 (G4)	Novel	SM10507, SM16360
Genomospecies 5 (G5)	*P. hibscicola*	SM1389, SM38795
Genomospecies 6 (G6)	Novel	SM3123

### Pan-genome analysis

**Fig. 4.**
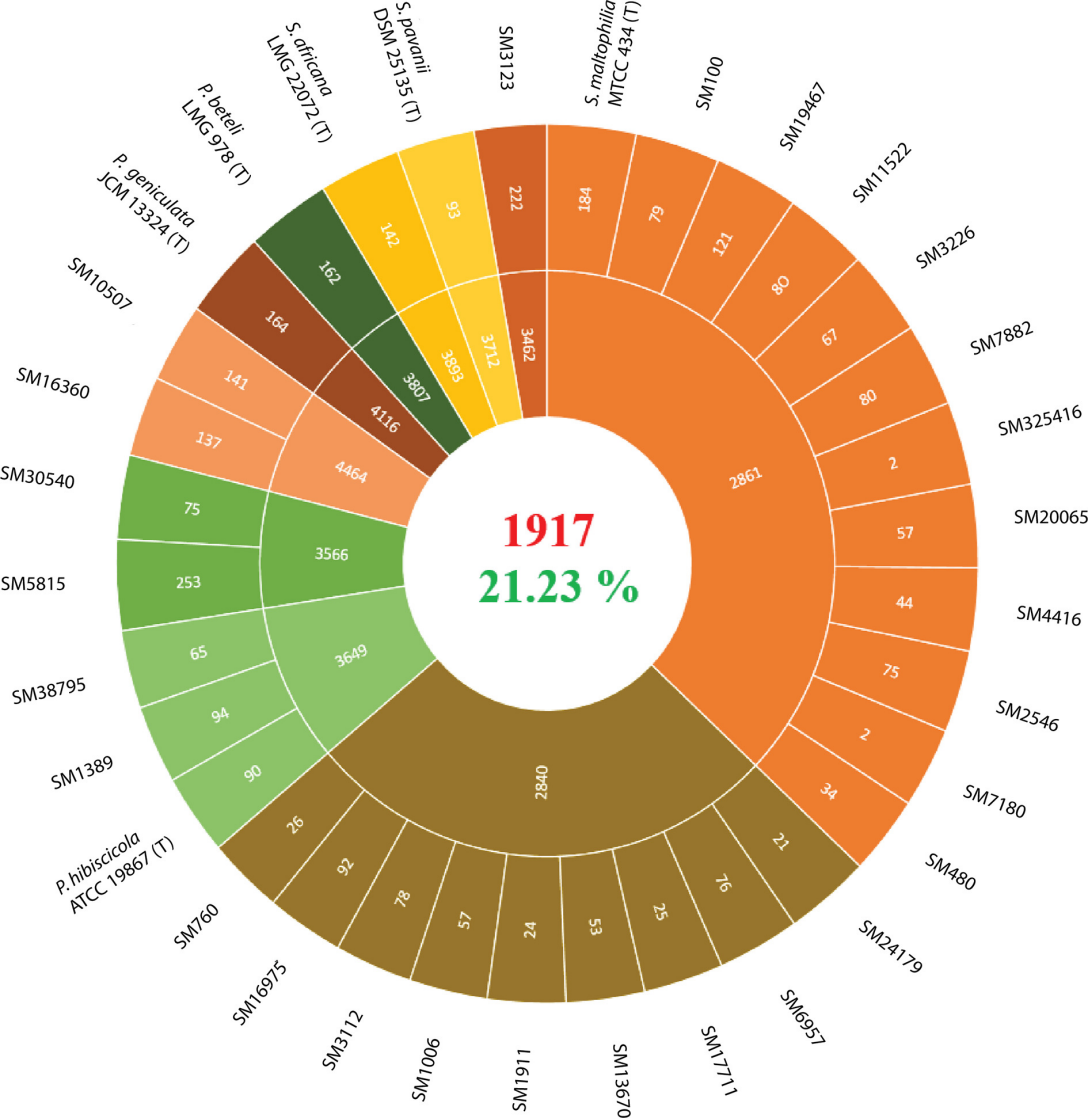
Number of orthologous CDSs belonging to the core, genomospecies-specific and strain-specific genes across the Smc. Strain names are given outside the circle. From outside to inside, the first and second circles represent the number of strain-specific CDSs and genomospecies-specific core-genome CDSs, respectively. The third circle at the centre represents the number of core genome CDSs of the Smc.

To obtain insight into the core genome, genomospecies-specific genes and strain-specific gene content, we performed pan-genome analysis of *S. maltophilia* genomospecies along with the type strains of Smc species. This analysis provided a measure of the intra-genomospecies variation in gene content. The orthologous CDSs shared among Smc members is 1917, which is ~21.23 % of the pan-genome size (9031 CDSs) (Fig. 4). The genomospecies-specific core genomes ranged from 2840 to 4464 CDSs, representing ~31 to ~49 % of the pan-genome size (Fig. 4). Core genome size is smaller than the group-wise core genomes. Among the group-wise core genome genomospecies, G2 (2840 CDSs) and G1 (2861 CDSs) have smaller core genomes due to a large number of genomes included in the analysis (Fig. 4). The strain-specific genes ranged from two to 253 CDSs, a widely variable genomic fraction (Fig. 4). Genomospecies G1 also had a small number of strain-specific genes, which is again in concordance with the fact that large numbers of the genomes were included in the analysis (Fig. 4).

**Fig. 5.**
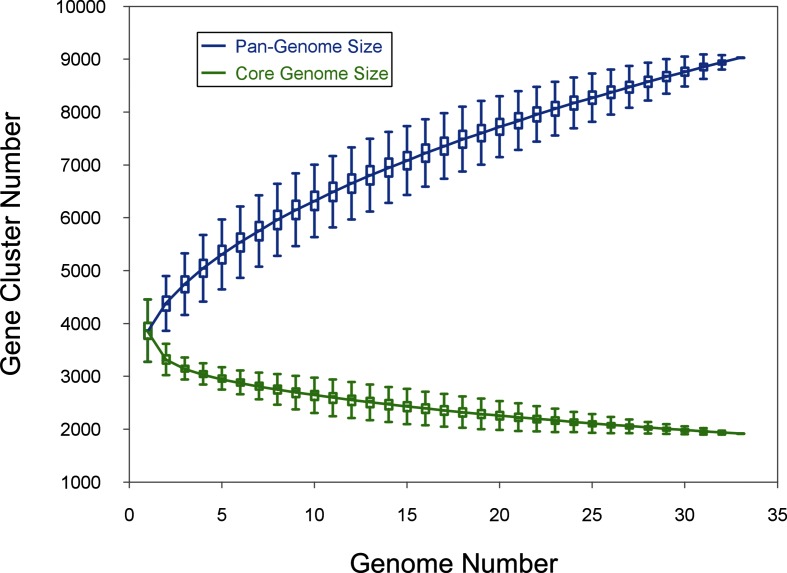
Pan and core genome analysis of *S. maltophilia* genomospecies. The size of *S. maltophilia* genomospecies pan-genome (blue) and shared gene clusters (green) are plotted as a function of the number of Smc genomes sequentially considered. The continuous curve represents calculated pan-genome size and the power-law regression model (*y*_pan_*=A*_pan_* x ^Bpan^+C*_pan_) was applied to the data. The best fit was obtained with *r*^2^=0.999, *A*_pan_=1344.45, *B*_pan_=0.45 and *C*_pan_=2525.33. The continuous curve represents the calculated core genome size and exponential curve fit model (*y*_core_*=A*_core_ *e ^Bcore.x^+C*_core_) was applied to the data. The best fit was obtained with *r*^2^=0.96755, *A*_core_=1775.67, *B*_core_=−0.08 and *C*_core_=1880.1. The pan and core genome size is 9031 and 1917, respectively.

The pan-genome plot (Fig. 5) clearly shows that even after the addition of all CDSs from 33 genomes, the plot is yet to reach a plateau and further addition of genomes will increase the pan-genome size. The power law regression model shows that the pan-genome of the Smc is ‘open’, as the γ-parameter value (*B*_pan_) is 0.45, and sequencing of isolates from the Smc is required to identify all genes of this complex. The core genome size decreases dramatically with the inclusion of each new genome, the curve almost approaches a plateau and further addition of new genomes may result in decreased core genome size (Fig. 5). Similar behaviour is observed in the plot of strain-specific CDSs against the number of genomes, the number of strain-specific CDSs gradually decreasing with the addition of new genomes (Fig. S2).

### Role of homologous recombination and mutations in diversification of clinical *S. maltophilia* isolates

To investigate the role of homologous recombination and mutations in the diversification of clinical isolates of *S. maltophilia*, we used the application ClonalFramML. The average relative rate of recombination (*R*) to mutation (θ) of the Smc was estimated to be *R*/θ=0.376066, mean DNA import length was δ=211 bp, and the mean divergence of imported DNA was ν=0.059. This suggests the occurrence of ~2.659 mutational events for each recombination event. The relative impact of recombination to mutation (*r*/*m*) is ~4.74 across the overall phylogeny of the Smc. To investigate the effect of recombination on the phylogenetic tree topology we used ClonalFrameML to reconstruct a more accurate phylogeny by removing the genomic divergence generated by recombination. Branch lengths of the ClonalFrameML tree were not consistent with the ML phylogeny, indicating the impact of recombination on the diversification of these isolates (Fig. S3).

### Resistome analysis

**Fig. 6.**
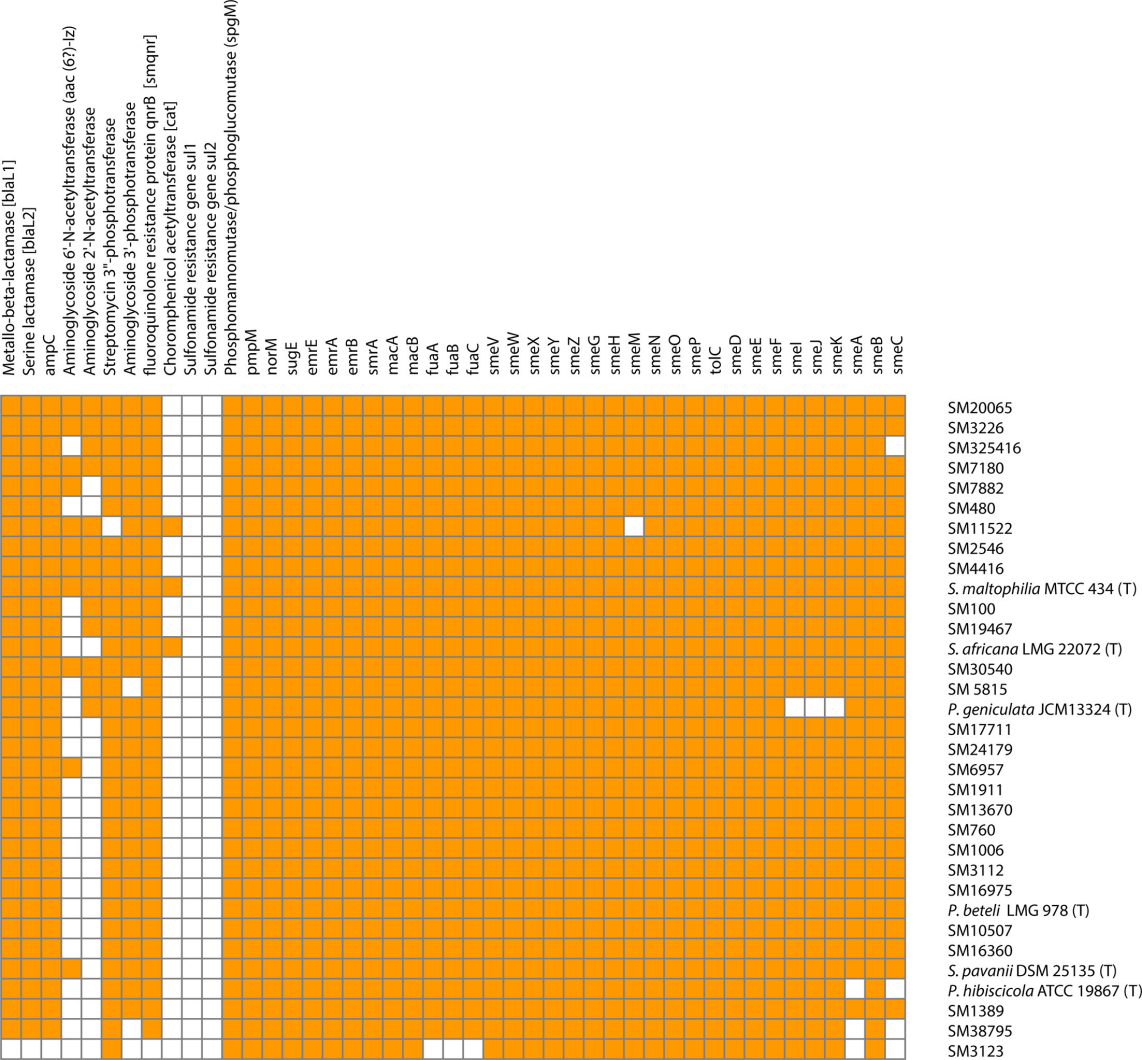
Distribution of antibiotic resistance and efflux pump genes across the Smc phylogeny. Gene names and strain names are labelled on the *x*-axis and *y*-axis of the heat map, respectively. Orange and white colours indicate the presence and absence of the gene in a particular isolate, respectively.

The resistome of a well-studied strain, *S. maltophilia* K279a, has been characterized and data on its drug resistance profile are available [24, 26]. We assessed the distribution of known antibiotic-resistant and efflux pump genes across various genomospecies of *S. maltophilia* clinical isolates (Fig. 6). The two chromosomally encoded β-lactamases *bla*_L1_ (Zn^2+^-dependent metalloenzyme) and *bla*_L2_ (serine β-lactamases), plus *amp*C, which are characteristics of *S. maltophilia* [7], are present in all isolates except SM 3123, which belongs to genomespecies G6 and does not harbour any of these β-lactamases. Resistance to the aminoglycosides group of antibiotics is mediated by aminoglycosidase-modifying enzymes such as aminoglycoside 6′-*N*-acetyltransferase (aac (6′)-Iz) [47], aminoglycoside 2′-*N*-acetyltransferase (aac (2′)-Iz) [24], aminoglycoside phosphotransferase (*aph* (3′)-IIc) [48] and streptomycin 3 phosphotransferase [24]. The distribution of aac (6′)-Iz and aac (2′)-Iz is limited to the Smc as they are present in six and 11 isolates, respectively, the majority of which belong to genomospecies G1. The streptomycin 3′-phosphotransferase and aminoglycoside phosphotransferase are present in all the isolates along with other members of the Smc but absent from isolates SM11522 SM38795, SM5815 and SM3123. The chloramphenicol acetyltransferase gene, *cat*, mediates resistance to chloramphenicol, which is exclusively present in *S. maltophilia* MTCC 434^T^ and SM11522. All strains except SM3123 carry the chromosomal *Smqnr* gene, which is responsible for resistance to quinolones. The gene *spgM*, involved in lipopolysaccharide biosynthesis and moderately involved in resistance to gentamicin, nalidixic acid, ceftazidime, piperacillin-tazobactam, polymyxin B, polymyxin E and vancomycin [49], is also present in the all isolates under study. The *sul* gene, which is responsible for resistance to the trimethoprim/sulfamethoxazole class of antibiotics [27], is not present in any of these isolates. There are five families of efflux pumps reported to be present in *S. maltophilia*: the resistance-nodulation-cell-division (RND) family, major facilitator superfamily (MFS), small multidrug resistance (SMR) family, ATP-binding cassette (ABC) family, and multidrug and toxic compound extrusion (MATE) family [25, 26], which are present in all the isolates under study with a few exceptions (Fig. 6). The well-characterized RND-type efflux pumps in the *S. maltophilia* genome are *smeABC*, *smeDEF*, *smeIJK*, *smeOP*, *smeVWX* and *smeYZ*. Apart from *smeABC*, the remaining RND-type efflux pumps are present in all the isolates under study. *smeABC* is absent from SM325416, SM38795, SM3123 and *P. hibiscicola* ATCC 19867. The *emrAB* efflux pump belonging to the MFS family is present in all the isolates under study, and confers resistance to hydrophobic antibiotics and compounds such as nalidixic acid, thiolactomycin and organomercurials [26]. SMR family pumps are considered responsible for resistance to β-lactams, macrolides, tetracyclines and quaternary ammonium compounds [50]. The *sugE* and *emrE* pumps are well-characterized SMR efflux pumps in *S. maltophilia* and are present in all the isolates. Two efflux pumps belonging to the ABC transporter family, *smrA* and *macAB*, were previously characterized from *S. maltophilia* and are present in all the isolates under study. The *smrA* pump is known to confer resistance to fluoroquinolones and tetracycline [50] and the *macABCsm* efflux pump confers intrinsic resistance to aminoglycosides, macrolides and polymyxins, which are present in all the isolates under study [51]. A unique tripartite fusaric acid efflux pump *fuaABC* responsible for fusaric acid resistance was reported in *S. maltophilia* [52], and is reported to be present in all isolates except SM3123. There are two genes, *pmp*M and *nor*M, encoding MATE efflux pumps [26], which are present in all the isolates and are known to be responsible for resistance to the quinolone family of drugs that includes ciprofloxacin, norfloxacin and ofloxacin.

## Discussion

*Stenotrophomonas* is a taxonomically challenging genus due to multiple taxonomic revisions in the past. *S. maltophilia* is an emerging opportunistic pathogen with high genetic diversity and is the only species in the genus that is known to be responsible for clinical infections. However, another species, *S. africana*, was isolated from human infections, and was later reclassified as *S. maltophilia* [53, 54]. Whole-genome sequencing of the type strains and historically associated reference strains revealed that *S. africana* represents another species of clinical importance [13]. Thus, there is a need to assess the intra-species diversity among clinical isolates of *S. maltophilia*. Advancements in sequencing technologies have enabled us to study the intra-species population structure based on genome sequence information [55]. Therefore, we carried out a whole-genome sequencing of 27 clinical isolates identified as *S. maltophilia* from a hospital located in northern India. Phylogenetic analysis using 16S rRNA and 23 housekeeping genes with the type strains of members of the genus *Stenotrophomonas* revealed that the clinical isolates were distributed exclusively over the Smc. This finding has implication for our understanding of the ecology of clinical *S. maltophilia* isolates within the genus, which is important for the utilization of other non-pathogenic members of the genus *Stenotrophomonas* for biotechnical purposes. Further phylogenomic and taxonogenomic analysis revealed the heterogeneous structure of the Smc. The current nomenclature suggests the presence of only two valid species (*S. maltophilia* and *S. pavanii*) and four misclassified species (*P. hibiscicola*, *P. geniculata*, *P. betele* and *S. africana*) belonging to the Smc. Our analysis suggests that there are six genomespecies among the clinical isolates of *S. maltophilia*; thus, the Smc should include at least ten distinct genomospecies. Genomospecies 1, which belongs to the core *S. maltophilia* group, is a dominant group (11/27 : 40.27 %) among sequenced isolates followed by Genomospecies 3, which is a putatively novel species (9/27 :  33.33 %) (Table 2). Two isolates from our study belong to *P. hibiscicola*, suggesting that *P. hibiscicola* is a putative novel species with an ability to cause human infections. This study also highlights the importance of type strain genomes in making accurate species assignments and in the discovery of novel species in the post-genomic era.

The pan-genome analysis suggests that the Smc has an open pan-genome and addition of newly sequenced genomes is required to identify all genes in the Smc. The small core genome size (21.23 %) suggests high genetic diversity and genomic heterogeneity among the isolates (Fig. 4). Further recombination analysis suggests that there is selection pressure acting on isolates of *S. maltophilia* for pathoadapation, which leads to the introduction of variations through homologous recombination and mutations. The impact of recombination is higher in the diversification of clinical *S. maltophilia* isolates because a single recombination event causes multiple nucleotide changes in the genome. However, the larger pan-genome, which is nearly five times larger than the core-genome, suggests that variation mediated by non-homologous gene transfer is also playing a role in the diversification of clinical *S. maltophilia* isolates. The novel genomospecies have a unique gene pool, which is different from *S. maltophilia*, suggesting that gene gain and loss events are shaping the genomes of clinical *S. maltophilia* isolates during the course of evolution.

Clinical isolates of *S. maltophilia* are well known for their high level of intrinsic resistance to most of the commonly used antibacterial agents, including β-lactams (cephalosporin, carbapenems), macrolides, fluoroquinolones, aminoglycosides, chloramphenicol, tetracyclines and polymyxins [7, 9, 25, 56]. In addition, the emergence of resistance against the treatment of choice, trimethoprim-sulfamethoxazole, is increasing [57, 58]. Along with intrinsic drug resistance genes, the multi-drug resistance phenotype is also mediated by intrinsically encoded efflux pumps [26]. The distribution of well-characterized antibiotic resistance and efflux pump genes of *S. maltophilia* across multiple novel genomospecies has provided insights into its antibiotic resistance capability. Further varying levels of resistant phenotype changes among these isolates can be correlated with point mutations and expression differences in resistant genes [59].

The identification of multiple genomospecies, which represent potential novel species of *Stenotrophomonas*, associated with human infections can serve as an important asset to clinicians. These clinical isolates of *S. maltophilia* are found to be considerably different from each other, despite originating from the same hospital. Further studies supplemented with polyphasic approaches are underway to ascertain if these putative genomospecies represent novel species. This information can be helpful for clinicians to manage infections caused by this clinically significant pathogen. Studies on the epidemiology, disease spectrum resistance and virulence traits for infections caused by putative novel species are required for species-specific diagnosis and treatment.

## Data bibliography

The 16S rRNA gene sequence of *Stenotrophomonas tumulicola* used in phylogenetic analysis.
1) Handa Y, Sugiyama J. GenBank. https://www.ncbi.nlm.nih.gov/nuccore/LC066089 (2008).
The Genomes used in the phylogenomic analysis and taxonogenomic analysis (detailed in the Table S1).
2) Patil PP, Midha S, Patil PB. GenBank. https://www.ncbi.nlm.nih.gov/nuccore/NZ_JALV00000000 (2016).
3) Patil PP, Midha S, Patil PB. GenBank. https://www.ncbi.nlm.nih.gov/nuccore/NZ_LLXW00000000.1 (2016).
4) Kyrpides N, Huntemann M, Han J, Chen A, Mavromatis K *et al.* GenBank. https://www.ncbi.nlm.nih.gov/nuccore/485091714?report=genbank (2013).
5) Patil PP, Midha S, Patil PB. GenBank. https://www.ncbi.nlm.nih.gov/nuccore/NZ_LLXV00000000.1(2016).
6) Patil PP, Midha S, Patil PB. GenBank. https://www.ncbi.nlm.nih.gov/nuccore/NZ_LDJN00000000.1 (2016).
7) Patil PP, Midha S, Patil PB. GenBank. https://www.ncbi.nlm.nih.gov/nuccore/NZ_LLXT00000000.1 (2016).
8) Patil PP, Midha S, Patil PB. GenBank. https://www.ncbi.nlm.nih.gov/nuccore/NZ_LDJK00000000.1 (2016).
9) Alavi P, Starcher MR, Thallinger G, Zachow C, Mueller H *et al*. GenBank. https://www.ncbi.nlm.nih.gov/nuccore/627787876?report=genbank (2014).
10) Patil PP, Midha S, Patil PB. GenBank. https://www.ncbi.nlm.nih.gov/nuccore/NZ_LLXU00000000.1(2016).
11) Patil PP, Midha S, Patil PB. GenBank. https://www.ncbi.nlm.nih.gov/nuccore/NZ_LDJH00000000.1 (2016).
12) Patil PP, Midha S, Patil PB. GenBank. https://www.ncbi.nlm.nih.gov/nuccore/NZ_LDJM00000000.1 (2016).
13) Patil PP, Midha S, Patil PB. GenBank. https://www.ncbi.nlm.nih.gov/nuccore/NZ_LDJO00000000.1 (2016).
14) Patil PP, Midha S, Patil PB. GenBank. https://www.ncbi.nlm.nih.gov/nuccore/NZ_LDJP00000000.1 (2016).
15) Patil PP, Midha S, Patil PB. GenBank. https://www.ncbi.nlm.nih.gov/nuccore/LLXS00000000 (2016).
16) Patil PP, Midha S, Patil PB. GenBank. https://www.ncbi.nlm.nih.gov/nuccore/NZ_LDJI00000000.1(2016).
17) Patil PP, Midha S, Patil PB. GenBank. https://www.ncbi.nlm.nih.gov/nuccore/NZ_LDJG00000000.1(2016).
18) Patil PP, Midha S, Patil PB. GenBank. https://www.ncbi.nlm.nih.gov/nuccore/NZ_LDJJ00000000.1 (2016).
19) Patil PP, Midha S, Patil PB. GenBank. https://www.ncbi.nlm.nih.gov/nuccore/NZ_LDJL00000000.1 (2016).
The well-characterised resistance genes were fetched from the genome of *S. maltophilia* K279a and used as a query in the BLAST analysis with sequenced *S. maltophilia* isolates.
20) Crossman LC, Gould VC, Dow JM, Vernikos GS, Okazaki A *et al*. GenBank. https://www.ncbi.nlm.nih.gov/nuccore/190572091?report=genbank (2008).

## Supplementary Data

Supplementary File 1
