# Supplementary material for: Taxonogenomics reveal multiple novel genomospecies associated with clinical isolates of Stenotrophomonas maltophilia
Source: Microb Genom. 2018 Aug 7;4(8):e000207. doi: 10.1099/mgen.0.000207 (PMC6159553; doi:10.1099/mgen.0.000207)
Supplement: Supplementary File 1 [file mgen-5-207-s001.pdf]

## Supplementary Information

**Figure S1**:

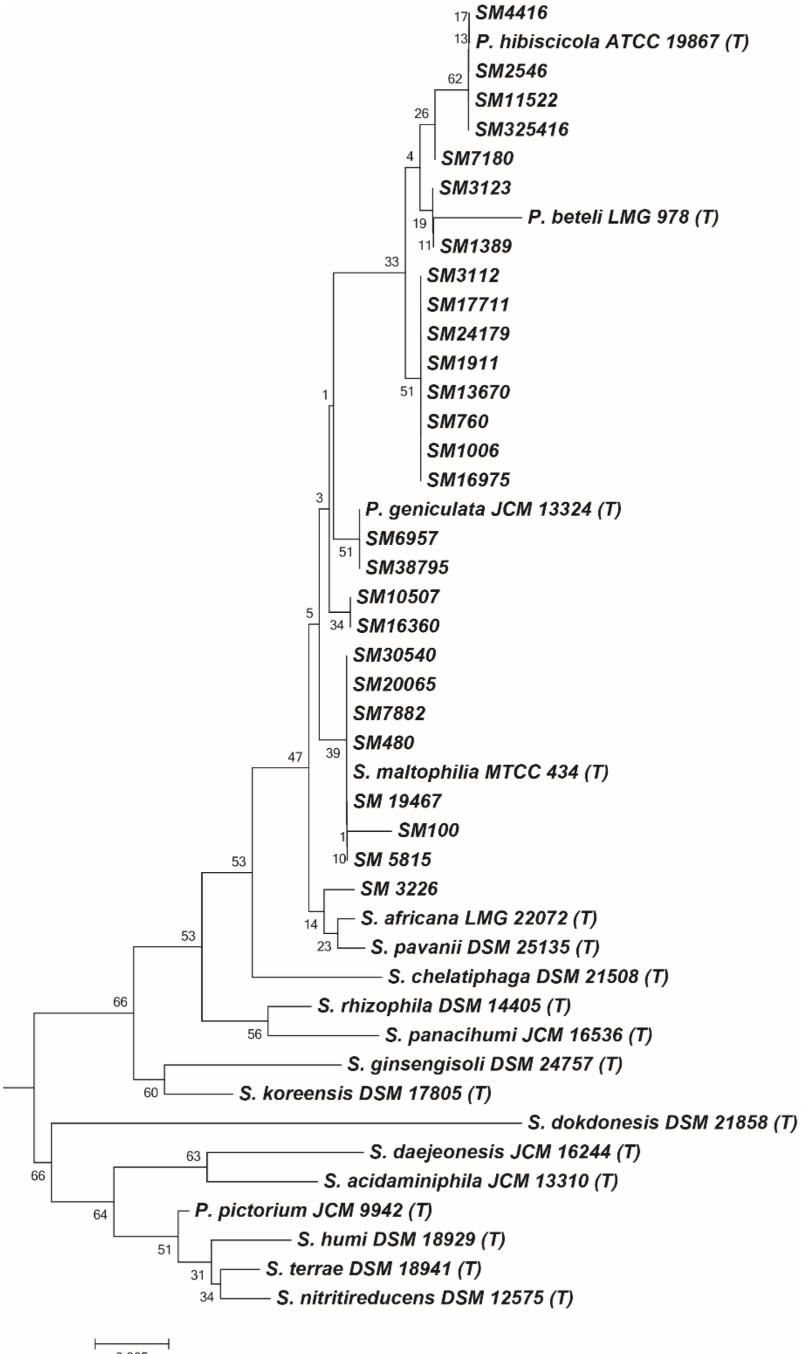


**Figure S1**: **Phylogenetic analysis of *S. maltophilia* clinical isolates within *Stenotrophomonas* genus.** Maximum likelihood reconstruction from complete 16S rRNA gene sequences.

**Figure S2:**

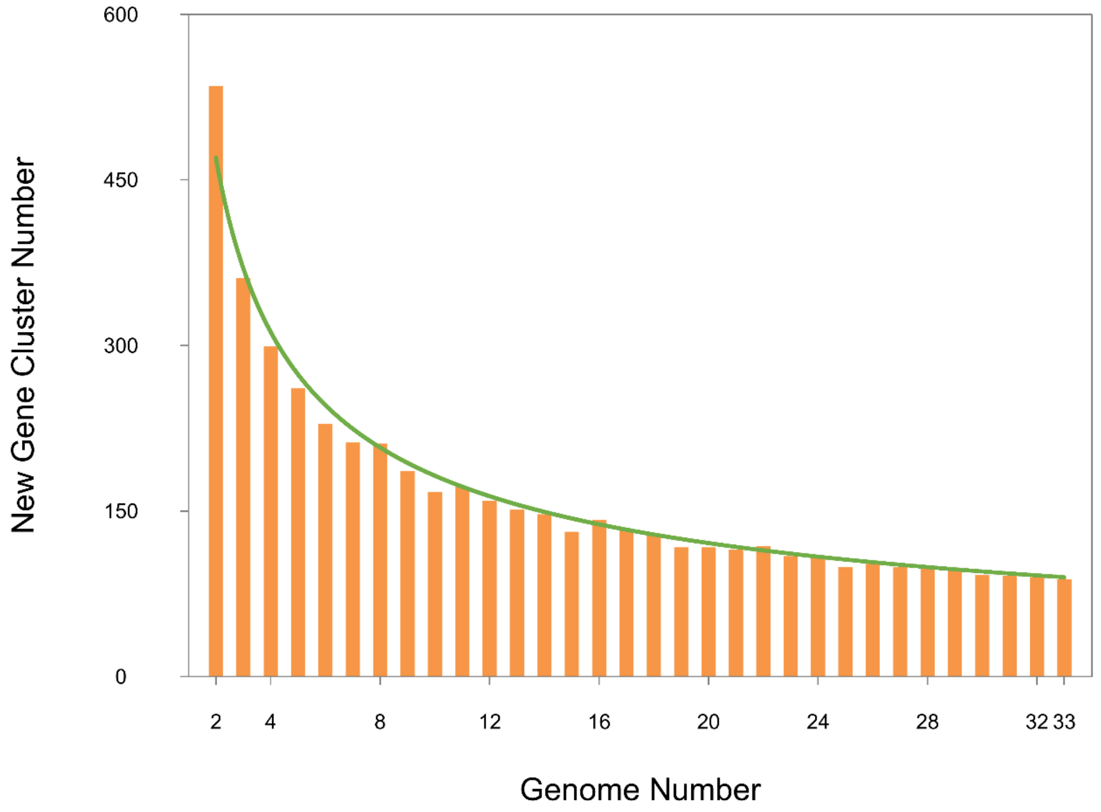


**Figure S2: New gene curve for the Pan-genome analysis**. Bars represent the number of new genes as a function of the sequentially added genome. The curve represents least squares fit for function $y = Ax^B$, where X= numbers of genome analyzed, with best fit obtained with a correlation $r^2$= 0.991715 for $A$ = 707.844, $B$ = -0.59.

**Figure S3:**

**A)** **B)**

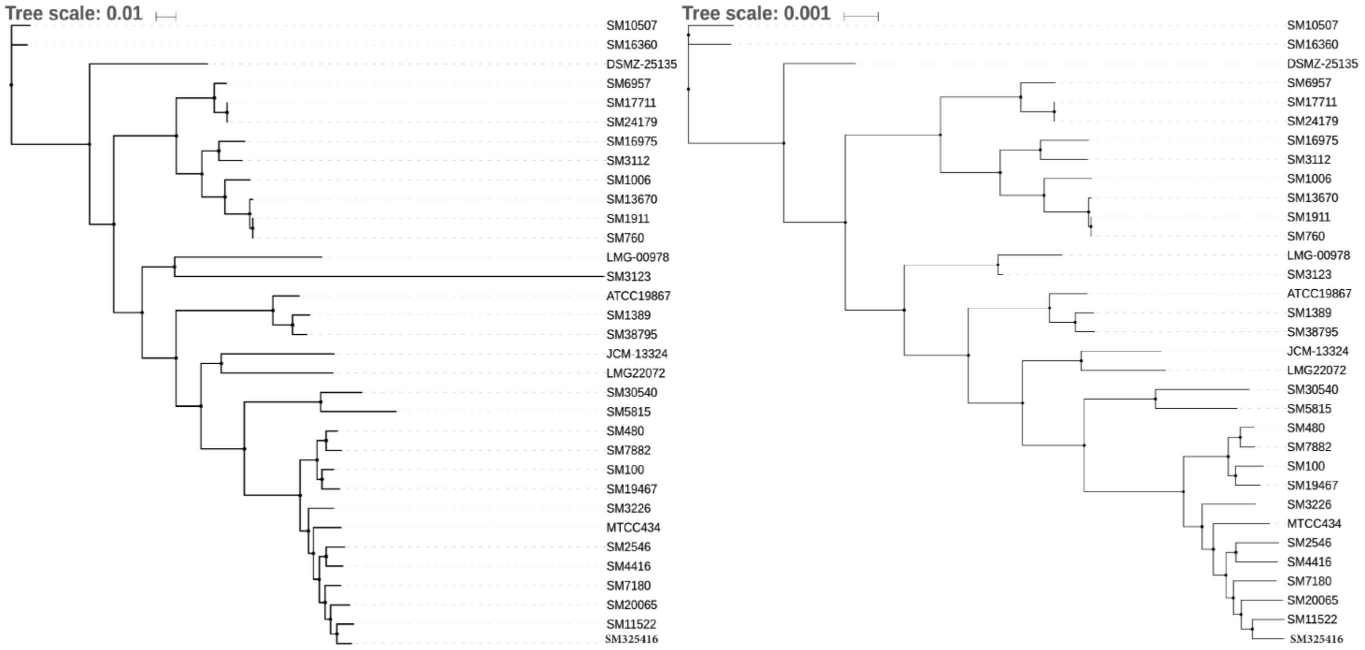


**Figure S3:**Maximum-likelihood reconstructed phylogeny comparison of (A) PhyML and B) ClonalFrameML phylogeny corrected for the recombination events.

**Table S1:**

The list of the genomes of the type strains of the genus *Stenotrophomonas* used in the phylogenetic and taxonogenomic analysis.

| Species | Accession No. | References |
|---|---|---|
| *S. maltophilia* MTCC 434$^{T}$ | JALV00000000 | Patil et. al.2016 |
| *S. africana* LMG 22072$^{T}$ | LLXW00000000 | Patil et. al.2016 |
| *P. hibiscicola* ATCC 19867$^{T}$ | ARNB01000000 | DOE-JGI (2013) |
| *P. beteli* LMG 978$^{T}$ | LLXV00000000 | Patil et. al.2016 |
| *S. pavanii* DSM 25135$^{T}$ | LDJN00000000 | Patil et. al.2016 |
| *P. geniculata* JCM*13324*$^{T}$ | LLXT00000000 | Patil et. al.2016 |
| *S. chelatiphaga* DSM 21508$^{T}$ | LDJK00000000 | Patil et. al.2016 |
| *S. rhizophila* DSM 14405$^{T}$ | CP007597 | Alavi et. al. 2014 |
| *S. panacihumi* JCM 16536$^{T}$ | LLXU00000000 | Patil et. al.2016 |
| *S. koreensis* DSM 17805$^{T}$ | LDJH00000000 | Patil et. al.2016 |
| *S. ginsengisoli* DSM 24757$^{T}$ | LDJM00000000 | Patil et. al.2016 |
| *S. acidaminiphila* JCM 13310$^{T}$ | LDJO00000000 | Patil et. al.2016 |
| *S. daejeonensis* JCM 16244$^{T}$ | LDJP00000000 | Patil et. al.2016 |
| *P. pictorium* JCM 9942$^{T}$ | LLXS00000000 | Patil et. al.2016 |
| *S. humi* DSM 18929$^{T}$ | LDJI00000000 | Patil et. al.2016 |
| *S. nitritireducens* DSM 12575$^{T}$ | LDJG00000000 | Patil et. al.2016 |
| *S. terrae* DSM 18941$^{T}$ | LDJJ00000000 | Patil et. al.2016 |
| *S. dokdonesis* DSM 21858$^{T}$ | LDJL00000000 | Patil et. al.2016 |
